# Case Reports of DRESS Syndrome and Symptoms Consistent with DRESS Syndrome Following Treatment with Recently Marketed Monoclonal Antibodies

**DOI:** 10.1155/2019/7595706

**Published:** 2019-06-09

**Authors:** James C. Di Palma-Grisi, Kesav Vijayagopal, Muhammad A. Muslimani

**Affiliations:** Università degli Studi di Pavia Facoltà di Medicina e Chirurgia, Piazza Volontari del Sangue Pal. Avis, Pavia (PV), 27100, Italy

## Abstract

**Background:**

Monoclonal antibodies constitute a potent and broadly tolerable drug class, representing for some conditions the first newly approved treatment in years. As such, many are afforded “fast-track” or “breakthrough therapy” designations by the U.S. Food and Drug Administration, leading to provisional approval before Phase III clinical trials are reported. Although these drugs are usually safe, some patients experience life-threatening complications—myositis and encephalitis have led to permanent or temporary recalls. Drug reaction with eosinophilia and systemic symptoms (DRESS) syndrome is a hypersensitivity condition easily missed due to its long incubation period and nonspecific presentation. This minireview is primarily intended as an abbreviated guide for practitioners who may be using these powerful treatments.

**Methodology:**

We searched PubMed using a string of symptoms consistent with DRESS syndrome and monoclonal antibodies approved by the FDA since 2015. Then, we excluded studies reporting dermatological complications of reactivation of nonherpetic infection, immunodeficiency-related infection, or reactions to the injection site or infusion. We searched for and accessed prior reviews and background studies via PubMed, Mendeley, and Google Scholar.

**Results:**

Two cases of DRESS syndrome were identified in the literature, both the result of treatment with daclizumab. There was one additional case of encephalitis without cutaneous symptoms caused by daclizumab. Drug-induced hypersensitivity dermatitis was reported following treatment with nivolumab and two cases of combination treatment with ipilimumab and either nivolumab or durvalumab produced maculopapular rash and bullae in the first patient and lichenoid dermatitis and blisters in the second patient.

**Conclusions:**

Daclizumab was the only recently approved monoclonal antibody associated with DRESS syndrome as such. Limitations in the diagnostic reliability of DRESS syndrome as a clinical entity and the lack of negative clinical trial reporting suggest enhanced vigilance on the part of clinicians and regulators may be warranted.

## 1. Introduction

Monoclonal antibodies are specific and effective treatments for immune system dysfunction, skin diseases, cancers and other conditions [1]. They can be synthesized as chimeric, humanized, or fully human antibodies—although fully human antibodies convey the least immunogenicity, adalimumab is capable of provoking antidrug antibody responses in some patients with rheumatoid arthritis [2]. Classical hypersensitivity reactions to monoclonal antibody therapy are broadly similar to those of other drugs. Manifestations of hypersensitivity reactions typically occur within six hours of drug administration and include cutaneous, cardiovascular, respiratory, gastrointestinal, and neurological changes [3].

Delayed hypersensitivity reactions include DRESS syndrome, Stevens-Johnson syndrome, vasculitis, and maculopapular eruptions, among other conditions [4]. Once the disease process has begun, patients typically develop pyrexia, diffuse rash, pruritis and papular, pustular, or vesicular erythema. Hematologic manifestations include lymphadenopathy, thrombocytopenia, and lymphocytosis, while eosinophilia is not universally present, a finding that has contributed to the multiple naming practices for the disease, such as “drug-induced hypersensitivity syndrome” [5].

Genetic factors appear to play a role and both diuretics and anticonvulsants appear particularly likely to cause DRESS syndrome. The mainstay of treatment is discontinuation of the offending drug and systemic glucocorticoids, although treatment may be confounded by multiple relapses after discontinuation [6]. The incubation period of DRESS syndrome is two to eight weeks, and the emergence of symptoms is thought to be connected to reactivation of latent human herpesvirus 6, although the exact mechanism behind this reactivation is unclear [7]. One proposed etiology is that the offending drugs or metabolites contribute to T-cell activation and viral reactivation [8].

Among monoclonal antibodies, a handful has been reported to cause DRESS syndrome, including several recently approved biologic agents. One reported case presented with widespread maculopapular exanthem and lymphadenopathy 54 days after treatment of basal cell carcinoma with vismodegib (approved 2012)—lymph node biopsy showed marked eosinophilic infiltrate in the absence of cancer cells [9]. A retrospective cohort study of patients treated with vemurafenib, a BRAF inhibitor, found among 131 patients, one case of DRESS syndrome, leading to permanent discontinuation of treatment [10]. Another case of DRESS syndrome occurred in a patient treated with sorafenib (approved 2013) for hepatocellular carcinoma, in which the authors report skin eruptions, eosinophilia, and both liver and kidney involvement [11].

Daclizumab, a monoclonal antibody approved on a “first in class” designation, indicating a novel mechanism of action, was voluntarily and permanently recalled in March 2018 due to multiple reports of encephalitis (FDA). Daclizumab was designed first as prophylaxis for acute organ rejection but was later repurposed as a treatment for relapsing multiple sclerosis. Its hepatotoxicity led to a “black box” warning label from the FDA. In the course of postmarketing pharmacovigilance, the European Medicines Agency (EMA) found that multiple patients developed eosinophilia, skin rash, and involvement of other organs or multiorgan failure suspected to be secondary to immunomodulation. These cases were only retroactively recognized to be DRESS syndrome [12]. More recent reports describe patients with symptoms overlapping meningoencephalitis and DRESS syndrome criteria [13].

Ipilimumab is a model drug approved in 2011 to inactivate cytotoxic T lymphocyte-associated antigen-4 (CTLA-4), a discovery whose importance was recognized by a Nobel prize in 2018. Yet the cutaneous manifestations of checkpoint inhibitors are varied and occasionally life-threatening. DRESS syndrome is one of the many adverse reactions associated with ipilimumab. Nivolumab and pembrolizumab target programmed cell death-1 (PD-1) and are known to cause adverse cutaneous reactions occurring later in treatment than CTLA-4 cutaneous reactions. Atezolizumab targets the PD-1 ligand (PD-L1) and causes similar cutaneous side effects as PD-1 inhibitors, but combination therapy presents a statistically significant increase in severe complications [14].

The risks of missing this clinical constellation are salient—the object of this mini-review is to assess the most recently FDA-approved monoclonal antibodies for cutaneous symptoms consistent with DRESS syndrome. Case studies offer detailed clinical pictures in which the presence or absence of DRESS syndrome can be relatively cleanly ruled in or out, while clinical trials offer aggregate data to assess the proportion of patients that develop symptoms consistent with DRESS syndrome.

## 2. Methods

Studies were included by searching PubMed using the following string: (dress OR erythema OR pruritus OR rash OR urticaria OR eruption) AND (secukinumab OR dinutuximab OR alirocumab OR evolocumab OR idarucizumab OR mepolizumab OR daratumumab OR necitumumab OR elotuzumab OR obiltoxaximab OR ixekizumab OR reslizumab OR atezolizumab OR daclizumab OR olaratumab OR bezlotoxumab OR brodalumab OR avelumab OR ocrelizumab OR dupilumab OR durvalumab OR sarilumab OR guselkumab OR “inotuzumab ozogamicin” OR benralizumab OR emicizumab OR ibalizumab OR tildrakizumab OR burosumab OR erenumab OR mogamulizumab OR lanadelumab OR “moxetumomab pasudotox” OR fremanezumab OR galcanezumab OR cemiplimab OR emapalumab OR ravulizumab OR caplacizumab). Studies were then excluded by agreement between all three authors if they did not include either (a) case studies of or (b) rates of dermatological adverse effects of a recently approved biologic. Studies reporting dermatological manifestations of (a) reactivation of suspected latent infection, (b) immunodeficiency-related infection, (c) only injection-site or infusion reactions fitting the search criteria, (d) cases identified as Stevens-Johnson syndrome or toxic epidermal necrolysis, or (e) cases reporting only pooled adverse effects were also then excluded. Prior reviews and background studies were searched via PubMed, Mendeley, and Google Scholar.

## 3. Syndromic Cutaneous Reactions

Total results by case report are arranged in Table 1. We found two cases of DRESS syndrome frankly identified as such in the literature, both as a result of daclizumab treatment of relapsing multiple sclerosis. In both cases, the syndrome was treated with high dose corticosteroids followed by plasmapheresis, but one case worsened and cyclophosphamide and rituximab were used in management [15]. There was also one case of drug-induced hypersensitivity dermatitis following treatment of metaplastic squamous cell carcinoma of the lung with nivolumab. This patient developed vesicles and bullae over 30% of his skin, which biopsy determined to be subepidermal bullous dermatitis with eosinophils. In the same case series, combination treatment with ipilimumab and nivolumab preceded a maculopapular rash and pruritis, followed a month later by bullae without mucosal involvement. A third patient treated with ipilimumab and durvalumab developed lichenoid dermatitis and pruritic, fluid-filled blisters on her foot, and a central pruritic rash [16]. There was another case of pruritus and erythematous macules and papules two months following treatment with durvalumab and tremelimumab, a combination checkpoint inhibitor regimen [17].

## 4. Nonsyndromic Cutaneous Reactions

Reports of nonsyndromic cutaneous reactions were also found following treatment with a handful of other recently approved monoclonal antibodies. Treatment with ixekizumab, an IL-17 blocker used to treat psoriasis, preceded new psoriasiform eruptions [18] in one patient, photosensitive cutaneous eruptions [19] in another, and lentiginous eruption [20] in a third patient. Treatment with secukinumab, an IL-17A blocker for treating psoriasis and ankylosing spondylitis, preceded one case of pemphigus [21] and another case of severe, ulcerative lichenoid mucositis [22]. Treatment with dupilumab, an IL-4 receptor blocker used to treat eczema, preceded three cases of erythema, pruritus, and widespread skin peeling [23]. Lastly, treatment with brodalumab preceded psoriaform hyperplasia and pustular lesions [24].

## 5. Discussion

DRESS syndrome is one distinct, severe, and idiosyncratic drug reaction that is more relevant than ever due to the rapid advancements in the production of novel biologics and their widespread implementation in the treatment of rheumatic, immunologic, and neoplastic diseases. Fortunately, DRESS syndrome is a rare condition, but the difficulty in diagnosing leads to an even lower rate of identification and reporting. Severe cutaneous reactions of any type reported in clinical trials are very rare, and the only reported cases of DRESS syndrome were in clinical trials of daclizumab [25]. DRESS syndrome poses risks of chronic sequelae and even fatality, which are further complicated by a prolonged latency period. The main challenge is to recognize the pathology for what it is, as most clinicians lack familiarity with the condition due to its very low incidence, its high variability among ethnicities, and the variations of its clinical presentation secondary to which organs are affected [26, 27]. While fever, rash, and eosinophilia are essential features for the diagnosis of this syndrome, some of the atypical features are dysphagia, agranulocytosis, and chylous ascites. The European Registry of Severe Cutaneous Adverse Reactions to Drugs and Collection of Biological Samples (RegiSCAR) criteria is widely used to make a diagnosis. RegiSCAR inclusion criteria for potential DRESS syndrome cases require at least 3 out of the following 4 signs: fever above 38°C, enlarged lymph nodes at a minimum of two sites, involvement of at least one internal organ, or blood count abnormalities [28]. Managing this syndrome mainly requires early removal of the causative agent and treatment with antihistamines and emollients in the mild form, corticosteroids in the moderate form, and plasmapheresis in the severe form [29].

Clinical manifestations are considered a hypersensitivity reaction to drugs when specific circulating antibodies and/or specifically sensitized lymphocytes are present. Pseudoallergic reactions on the other hand occur when manifestations similar to those of a hypersensitivity reaction are observed in the absence of immunological specificity [30, 31]. Although this pathophysiological classification is generally useful, it does not adequately allow inferring, based on clinical symptoms, in which immune mechanism is involved, as occurring in toxic epidermal necrolysis, Stevens-Johnson syndrome, and DRESS syndrome [32]. The available drug presentation models that explain how small drug antigens might interact with HLA and T cell receptor (TCR) molecules in drug hypersensitivities include the hapten theory, the p-i concept, the altered peptide repertoire model, and the altered TCR repertoire model. The broad spectrum of clinical manifestations of drug hypersensitivity regardless of the involved drug, as well as the diverse pathological mechanisms involved, makes diagnosis and management more challenging [33].

In nonclinical toxicity studies, type I hypersensitivity reactions are often observed following administration of biologics [34]. Type I reactions are mediated by specific IgE antibodies associated with mast cells and basophils, and their clinical manifestations may include anaphylaxis or urticaria/angioedema. Another mechanism suspected to be a potential consequence of immunostimulation by biologics is through the occurrence of more frequent hypersensitivity reactions to unrelated allergens [35]. Eosinophilic activation as well as activation of the inflammatory cascade in DRESS syndrome may be induced by interleukin-5 release from drug-specific T-cells [36]. A challenge remains to identify the optimal balance between dosage, benefits, and side effects, which could sometimes be more serious than the treated disease. Latent hypersensitivity reactions and viral reactivations in susceptible populations should warrant a thorough revision of the safety profiles of culprit biologics.

In contrast to an overall low incidence in clinical trials, there is a plethora of documented cases of severe cutaneous reactions, mimicking those encompassed by the clinical picture of DRESS syndrome, as a result of treatment with various biologics. Revising the literature, it is observed that some biologics caused hypersensitivity reactions independent of the dose and the duration of the therapy. Other biologics caused hypersensitivity reactions that seem to correlate with the dose, total number of cycles, and/or spacing between cycles (e.g., dinutuximab, necitumumab, and ixekizumab). Interestingly the hypersensitivities accompanying mogamulizumab-kpkc administration were all categorized as acute infusion reactions. The only incidence where DRESS was namely reported is in 0.1% of patients receiving 150mg of subcutaneous daclizumab every 4 weeks for 96 weeks. The reported overall cutaneous adverse events were significantly high for necitumumab. Molecularly, necitumumab's large paratope cavities are speculated to make it less susceptible to resistance through EGFR epitope mutation [37], a characteristic which could theoretically be a contributing factor to latent cutaneous adverse effects. In treatment with daclizumab, observed cutaneous adverse events are likely related to the immunomodulatory effects it exerts on innate lymphoid cells, including natural killer cells [38]. Identifying the mechanisms by which each biologic produces these severe side effects is crucial to generating appropriate countermeasures, including allergic prescreening, prophylactic regimens, pharmacovigilance, early-response drugs, and supportive care measures.

While multiple studies have estimated the mortality rate of DRESS syndrome to be approximately 10%, the severity and extent of cutaneous involvement do not always correlate with the extent of internal organ involvement [39, 40]. The underreported incidents of DRESS syndrome complication may arise from a primary failure to recognize its syndromic appearance and to correlate it to biologic administration, especially in cases when the latter had been discontinued. Given the difficulties encountered while attempting to diagnose DRESS syndrome, it is justifiable to have doubts as to whether all cases consistent in clinical review were properly identified as DRESS syndrome. It is a valid assumption that a group of relevant cutaneous symptoms reported individually amounts to an acceptable diagnosis of DRESS syndrome. Most notably the timing of the manifestations is critical for the correct diagnosis [41]. Nonetheless all confirmed cases of hypersensitivity reactions should be reported in detail, as postmarketing surveillance and risk minimization requirements for biologics are set in place to ensure that long-term, real-world safety data are collected to assess biologics in clinical practice [42].

The diagnostic tools, including skin testing and in vitro testing, to evaluate for immediate hypersensitivity reactions remain insufficient [43]. Santiago et al. resorted to evaluating the safety and efficacy of patch testing in DRESS syndrome, thus attempting to identify a drug-dependent delayed hypersensitivity mechanism, but its application remains limited to DRESS syndrome induced by antiepileptic drugs [44]. Genotyping for HLA markers can be used as a screening tool before prescribing such potent drugs and can therefore prevent or at least mitigate DRESS syndrome in specific populations [45].

## 6. Conclusion

DRESS syndrome as such is very rare, but may be significantly underreported due to its complicated presentation. Given that the evidence for hypersensitivity reactions and immune dysregulation from biologics is not lacking, allergists, immunologists, and dermatologists should be involved in managing these patients to achieve optimal care. Certain genetic markers have high sensitivity and specificity, providing a plausible basis for the future development of tests to identify individuals at risk for biologic hypersensitivity.

## Figures and Tables

**Table 1:** Full results of the case study screen organized by reporter and monoclonal antibody.

Reporter	Monoclonal Antibody	Cutaneous Adverse Effect
Rauer et. al.	Daclizumab	DRESS syndrome

	Daclizumab	DRESS syndrome

Naidoo et. al.	Nivolumab	Drug-induced hypersensitivity dermatitis

	Ipilimumab/Nivolumab	Maculopapular rash, vesicles, erosions

	Ipilimumab/Durvalumab	Lichenoid dermatitis, blisters, exanthema

Fontecilla et. al.	Durvalumab/Tremelimumab	Epidermal necrolysis, eosinophilic infiltrate

Oiwa et. al.	Ixekizumab	Psoriasiform eruptions

Anthony et. al.	Ixekizumab	Pruritic, photosensitive plaques

Maria et. al.	Ixekizumab	Lentiginous eruption

Hayashida et. al.	Secukinumab	Pemphigus vulgaris

Thompson et. al.	Secukinumab	Ulcerative lichenoid mucositis

Al Hammadi & Parmar	Dupilumab	Erythema, pruritus and diffuse skin peeling

Takahashi et. al.	Brodalumab	Palmar pustular eruption
